# High Prevalence and Onward Transmission of Non-Pandemic HIV-1 Subtype B Clades in Northern and Northeastern Brazilian Regions

**DOI:** 10.1371/journal.pone.0162112

**Published:** 2016-09-07

**Authors:** Flavia Divino, Andre de Lima Guerra Corado, Felipe Gomes Naveca, Mariane M. A. Stefani, Gonzalo Bello

**Affiliations:** 1 Laboratório de AIDS e Imunologia Molecular, Instituto Oswaldo Cruz, FIOCRUZ, Rio de Janeiro, RJ, Brazil; 2 Instituto Leônidas e Maria Deane, Fundação Oswaldo Cruz, Manaus, AM, Brazil; 3 Tropical Pathology and Public Health Institute, Federal University of Goiás, Goiânia, GO, Brazil; National Institute of Health, ITALY

## Abstract

The Human immunodeficiency virus type-1 (HIV-1) epidemic in Brazil is mainly driven by the subtype B pandemic lineage (B_PANDEMIC_), while Caribbean non-pandemic subtype B clades (B_CAR_) seem to account for a very low fraction of HIV-infections in this country. The molecular characteristics of the HIV-1 subtype B strains disseminated in the Northern and Northeastern Brazilian regions, however, have not been explored so far. In this study, we estimate the prevalence of the HIV-1 B_PANDEMIC_ and B_CAR_ clades across different Brazilian regions and we reconstruct the spatiotemporal dynamics of dissemination of the major Brazilian B_CAR_ clades. A total of 2,682 HIV-1 subtype B *pol* sequences collected from 21 different Brazilian states from the five country regions between 1998 and 2013 were analyzed. Maximum Likelihood phylogenetic analyses revealed that the B_CAR_ strains reached 16 out 21 Brazilian states here analyzed. The B_CAR_ clades comprise a low fraction (<10%) of subtype B infections in most Brazilian states analyzed, with exception of Roraima (41%), Amazonas (14%) and Maranhão (14%). Bayesian phylogeographic analyses indicate that B_CAR_ strains originally from the Hispaniola and Trinidad and Tobago were introduced at multiple times into different states from all Brazilian regions and a few of those strains, probably introduced into Roraima, Maranhão and São Paulo between the late 1970s and the early 1980s, established secondary outbreaks in the Brazilian population. These results support that the HIV-1 subtype B epidemics in some Brazilian states from the Northern and Northeastern regions display a unique molecular pattern characterized by the high prevalence of B_CAR_ lineages, which probably reflects a strong epidemiological link with the HIV-1 epidemics in the Caribbean region.

## Introduction

According to estimations of the Brazilian Ministry of Health, about 780,000 people were living with the Human Immunodeficiency Virus Type 1 (HIV-1) in Brazil at 2014 [1]. Most Brazilian AIDS cases notified in the 2000–2015 period were concentrated in the Southeastern region (48%), followed by the Southern (22%), Northeastern (17%), Northern (7%) and Central-Western (6%) regions [1]. The AIDS Brazilian epidemic is primarily driven by the HIV-1 subtype B, followed by subtypes F1, C, and recombinant forms among those subtypes, although the relative prevalence of different HIV-1 genetic variants greatly vary across Brazilian regions [2–7].

Subtype B is the most prevalent HIV-1 lineage circulating in the Americas and its dissemination was probably initiated by the introduction of a founder strain from Central Africa into Haiti around the middle 1960s [8]. Between the late 1960s and the early 1970s, the subtype B seems to have moved out from the island of Hispaniola (shared by Haiti and the Dominican Republic) on several independent occasions, reaching the United States (US) and some neighboring Caribbean countries [8]. One subtype B variant introduced in the US was successfully disseminated within this country and to other countries around the world, establishing a pandemic clade (B_PANDEMIC_) [8]. Other subtype B variants, by contrast, remained mostly restricted to the Caribbean region and established a number of non-pandemic Caribbean clades (B_CAR_) [8,9].

The non-pandemic B_CAR_ lineages account for an important fraction of HIV-1 subtype B infections in several American countries including: Haiti and the Dominican Republic (~75%), Jamaica (~50%), Trinidad and Tobago (~95%), other Lesser Antilles (~40–75%), French Guiana (56%) and Suriname (54%) [9,10]. The non-pandemic B_CAR_ strains have been also disseminated from the Caribbean into several Latin American countries [10–12], with evidence of onwards transmission in Argentina, Brazil, Mexico, Panama and Venezuela [10,12]. Those secondary outbreaks established in Latin America, however, were of small size and the B_CAR_ strains only account for a minor fraction (<10%) of HIV-1 subtype B infections in that region [10,12].

A previous study conducted by our group, estimated that B_CAR_ strains only explain 1.7% of subtype B infections in Brazil [10]. Most Brazilian subtype B sequences used in that previous study, however, were from the Southeastern, Southern and Central-Western country regions. The objective of this study was to estimate the relative prevalence of the B_PANDEMIC_ and B_CAR_ clades in all Brazilian regions and to reconstruct the spatiotemporal dynamics of dissemination of the HIV-1 B_CAR_ clades circulating in the country. For this, we used a comprehensive dataset of HIV-1 subtype B *pol* sequences (*n* = 2,682) isolated from 21 different Brazilian states from the five country regions between 1998 and 2012. Brazilian HIV-1 subtype B sequences were combined with reference sequences of the B_PANDEMIC_ and the B_CAR_ clades and then subjected to Maximum Likelihood and Bayesian phylogeographic analyses.

## Materials and Methods

### Brazilian HIV-1 subtype B *pol* sequence dataset

We downloaded all HIV-1 subtype B *pol* sequences from Brazil with information about sampling state and that covered the entire protease and partial reverse transcriptase (PR/RT) regions (nucleotides 2253–3260 relative to HXB2 clone), available at the Los Alamos HIV Database (http://www.hiv.lanl.gov) by September 2015. Only one sequence per subject was selected and those sequences with incorrect subtype assignment were removed. These sequences were combined with Brazilian HIV-1 subtype B *pol* sequences from the Northern region recently published (*n* = 318) [7,13] and others that were newly generated (*n* = 71). New HIV-1 subtype B *pol* sequences were obtained from HIV-1-infected persons that attended the Public Health Central Laboratory from Roraima (LACEN-RR) in 2013. Blood samples were transported to the Instituto Leônidas e Maria Deane (FIOCRUZ) in Manaus for HIV amplification and subtyping as described previously [13]. All patients were informed of the procedures and signed the informed consent. The study was approved by the Ethics Committee of the "Universidade Federal de Roraima" (CAAE 15629013.8.0000.5302). This resulted in a final data set of 2,682 subtype B *pol* sequences isolated from 21 Brazilian states distributed across the five country regions (Table 1). The subtype assignment of all sequences was confirmed using the REGA HIV subtyping tool v.2 [14] and by performing phylogenetic analyses (see below) with HIV-1 group M subtype reference sequences.

**Table 1:** HIV-1 subtype B *pol* (PR/RT and RT) Brazilian sequences.

Region	State	Code	Public database/ Published	Newly generated	Sampling time
Southern	Paraná	PR	50	-	2001–2009
	Rio Grande do Sul	RS	138	-	1998–2009
	Santa Catarina	SC	20	-	2005–2009
Southeastern	Espírito Santo	ES	59	-	1997
	Minas Gerais	MG	69	-	2002–2010
	Rio de Janeiro	RJ	179	-	2002–2010
	Sao Paulo	SP	1,205	-	1998–2010
Central-Western	Goiás	GO	150	-	2003–2010
	Mato Grosso	MT	64	-	2008–2009
	Mato Grosso do Sul	MS	38	-	2008–2010
Northeastern	Bahia	BA	14	-	2009
	Maranhão	MA	70	-	2012
	Pernambuco	PE	97	-	2009–2010
	Piauí	PI	72	-	2011–2012
Northern	Acre	AC	11	-	2010–2011
	Amapá	AP	73	-	2013
	Amazonas	AM	104	-	2009–2011
	Pará	PA	89	-	2010–2011
	Tocantins	TO	46	-	2008
	Rondônia	RO	32	-	2010–2011
	Roraima	RR	31	71	2010–2013

### Phylogenetic analysis

HIV-1 Brazilian sequences were aligned with subtype B *pol* (PR/RT) sequences from the US (*n* = 165), France (*n* = 135) and the Caribbean (*n* = 279) representative of the B_PANDEMIC_ and the B_CAR_ clades described previously [9,12]. Sequences were aligned using the Clustal W program [15] and all sites associated with major antiretroviral drug resistance in PR and RT were excluded. Maximum Likelihood (ML) phylogenetic trees were inferred under the GTR+I+Γ nucleotide substitution model selected using the jModeltest program [16]. The ML trees were reconstructed with the PhyML program [17] using an online web server [18]. Heuristic tree search was performed using the SPR branch-swapping algorithm and the reliability of the obtained topology was estimated with the approximate likelihood-ratio test (*aLRT*) [19] based on the Shimodaira-Hasegawa-like procedure. The ML trees were visualized using the FigTree v1.4.0 program [20].

### Analysis of the spatiotemporal dispersion pattern

The evolutionary rate, the age of the most recent common ancestor (*T*_MRCA_) and the spatial diffusion pattern of HIV-1 B_CAR_ clades circulating in Brazil were jointly estimated using the Bayesian Markov Chain Monte Carlo (MCMC) approach as implemented in BEAST v1.8 [21,22] with BEAGLE to improve run-time [23]. Analyses were performed using the GTR+I+Γ_4_ nucleotide substitution model, a relaxed uncorrelated lognormal molecular clock model [24], and a Bayesian Skyline coalescent tree prior [25]. The mean evolutionary rates previously estimated for the subtype B *pol* gene (2.0–3.0 x 10^−3^ subst./site/year) [12,26–28] were incorporated as an informative prior interval. Migration events throughout the phylogenetic history and the most relevant migration pathways were reconstructed using a reversible discrete phylogeography model and the Bayesian stochastic search variable selection (BSSVS) approach [29], with a CTMC rate reference prior [30]. Three MCMC chains were run for 500 x 10^6^ generations and then combined using LogCombiner v1.8. Convergence and uncertainty of parameter estimates were assessed by calculating the Effective Sample Size (ESS) and 95% Highest Probability Density (HPD) values, respectively, after excluding the initial 10% of each run with Tracer v1.6 [31]. The maximum clade credibility (MCC) tree was summarized with TreeAnnotator v1.8 and visualized with FigTree v1.4.0. Migratory events and Bayes factor rates were summarized using the cross-platform SPREAD application [32].

### Nucleotide Sequence Accession Numbers

HIV-1 subtype B *pol* (PR/RT) sequences from Roraima were deposited in GenBank under accession numbers KX443015-KX443025, KX443027-KX443059 and KX443061- KX443087.

## Results

### Prevalence of the HIV-1 B_PANDEMIC_ and B_CAR_ clades in Brazil

**Fig 1:**
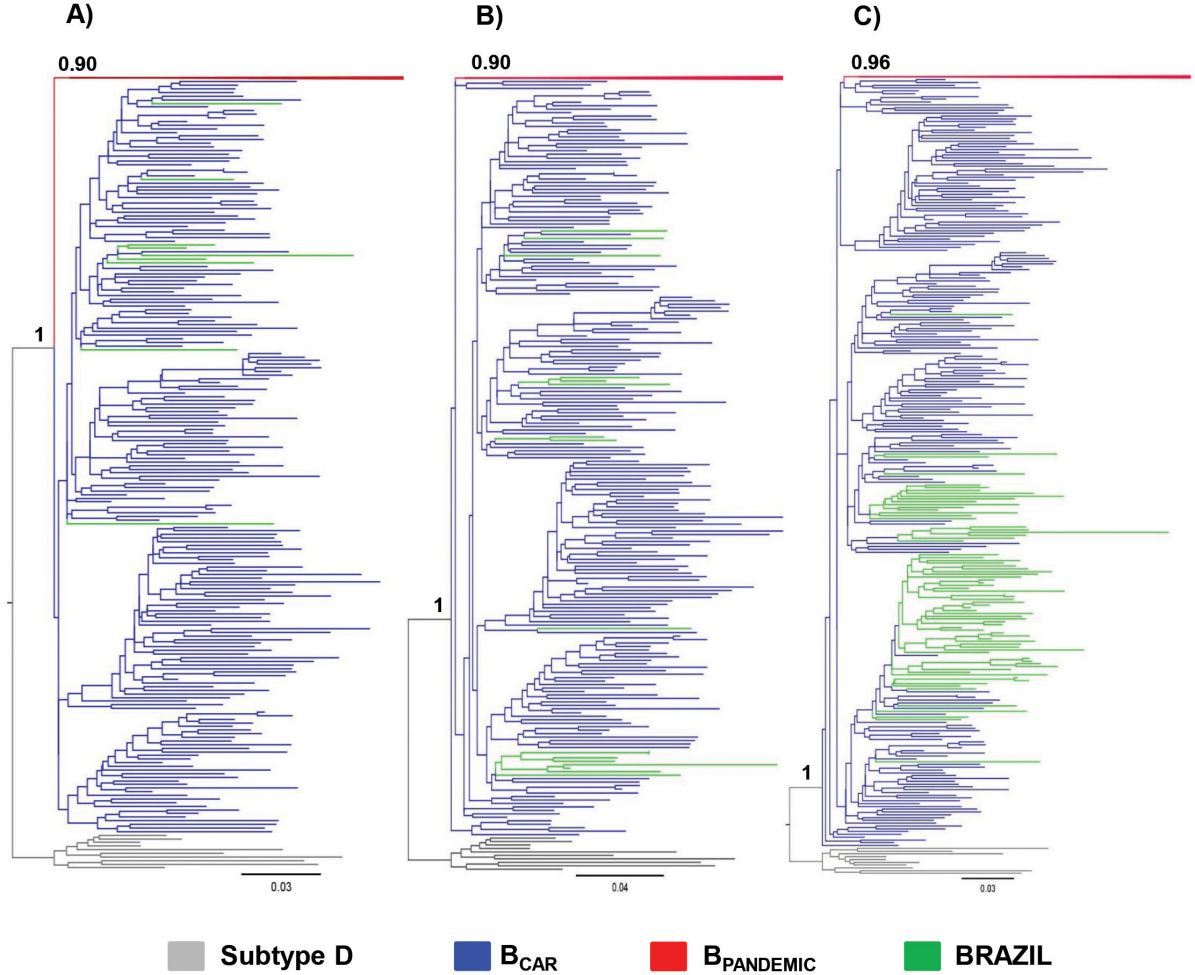
ML phylogenetic tree of HIV-1 subtype B *pol* PR/RT sequences (~1,000 nt) circulating in Brazil (*n =* 2,682) and representative sequences of the B_PANDEMIC_ (*n* = 300) and the B_CAR_ (*n* = 200) clades. Brazilian subtype B *pol* sequences were subdivided in three subsets according to their geographic origin: A) sequences from Sao Paulo, B) sequences from the Central-Western/Southern/Southeastern (except Sao Paulo) regions, and C) sequences from the Northern/Northeastern regions. Branches are colored according to the geographic origin/clade classification of each sequence as indicated at the legend at bottom. The B_PANDEMIC_ clade was collapsed for visual clarity. The a*LRT* support values are indicated at key nodes. Trees were rooted using HIV-1 subtype D reference sequences. The branch lengths are drawn to scale with the bar at the bottom indicating nucleotide substitutions/site.

**Fig 2:**
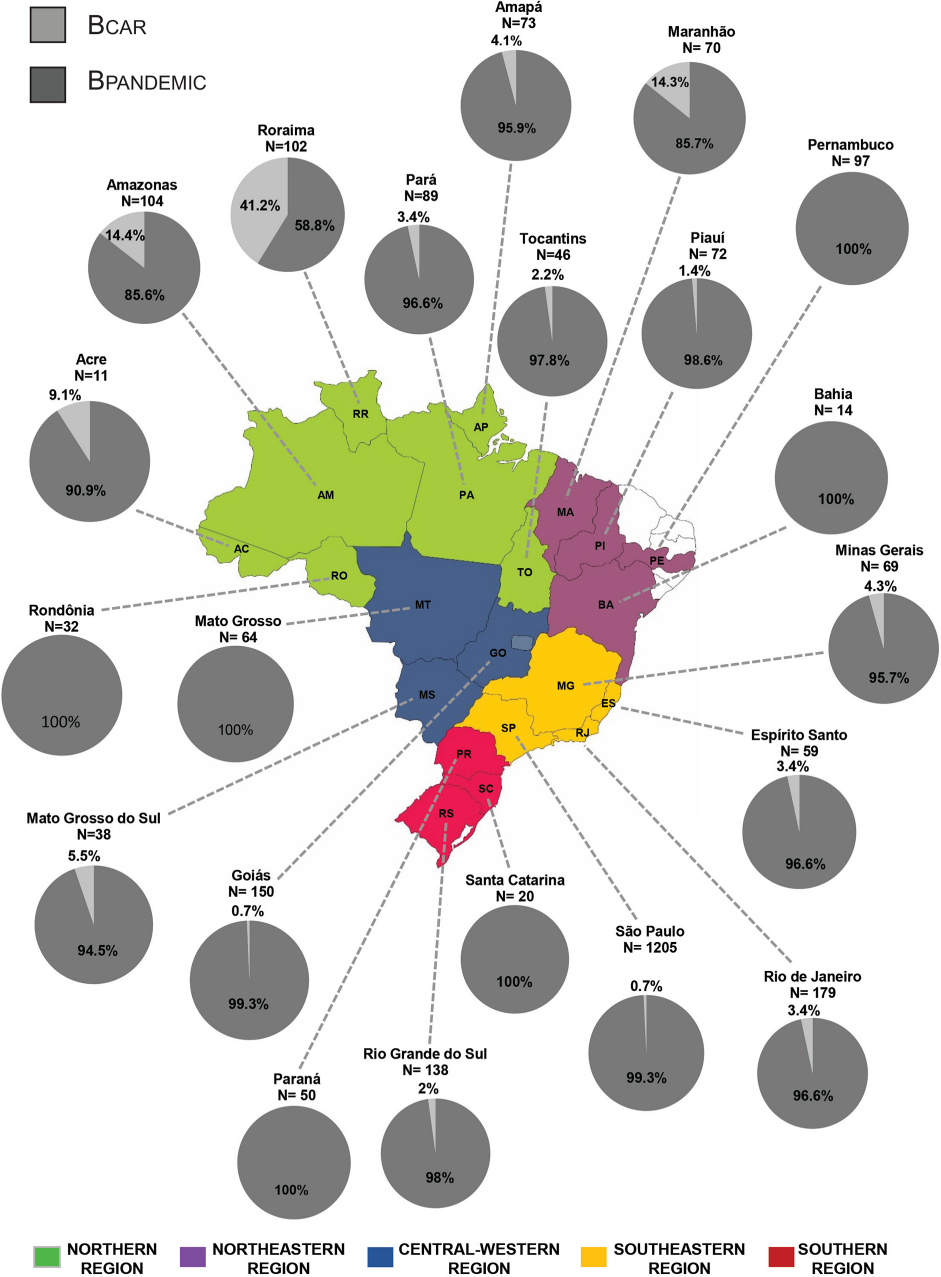
Estimated proportion of B_CAR_ and B_PANDEMIC_ clades among HIV-1 subtype B infected individuals from different Brazilian states. The total number of sequences analyzed in each locality is indicated. States were colored according to the Brazilian region of origin as indicated in the legend at bottom, with exception of those states with no HIV-1 sequences included in this study (white). Map was created from a template obtained from d-maps.com (http://d-maps.com/carte.php?numcar=4843&lang=en).

A total of 2,682 HIV-1 subtype B *pol* sequences isolated from 21 Brazilian states from the Southeastern (*n* = 1,512), Northern (*n* = 457), Northeastern (*n* = 253), Central-Western (*n* = 252) and Southern (*n* = 208) regions were analyzed in this study (Table 1). Brazilian HIV-1 subtype B *pol* sequences were divided in three subsets and each subset was combined with a reference dataset containing 500 B_PANDEMIC_ sequences from the US and France and 200 B_CAR_ sequences from the Caribbean, selected from a previous study [9] (S1 Table). The ML analyses of all three subsets confirmed that B_PANDEMIC_ reference sequences branched in a highly supported (aLRT > 0.90) monophyletic clade nested within basal B_CAR_ reference sequences (Fig 1). These analyses also showed that B_CAR_ sequences were detected in 16 out 21 Brazilian states here analyzed, although with highly variable prevalence across locations (Fig 2). The B_CAR_ sequences account for a very large proportion (41%) of HIV-1 subtype B infections in the state of Roraima, a relative large/moderate proportion (14%) in the states of Amazonas and Maranhão, and a low proportion (<5%) in the remaining Brazilian states. When analyzed by Brazilian region, the highest proportion of B_CAR_ sequences was observed in the Northern (17%), followed by the Northeastern (4%), Central-Western (1%), Southeastern (1%) and Southern (1%) regions. Analysis of the epidemiological characteristics of the HIV-1 B_CAR_-infected patients reveals that most individuals were male (58%), and that the heterosexual mode of transmission was the predominant one (65%), followed by men having sex with men (MSM, 23%). Diagnosis of HIV-1 infection ranged between 1995 and 2013 and the country of origin of all individuals was Brazil, with exception of one individual from Guyana that attended the Public Health Central Laboratory from Roraima.

### Dispersal pattern of the HIV-1 B_CAR_ strains from the Caribbean into Brazil

**Fig 3:**
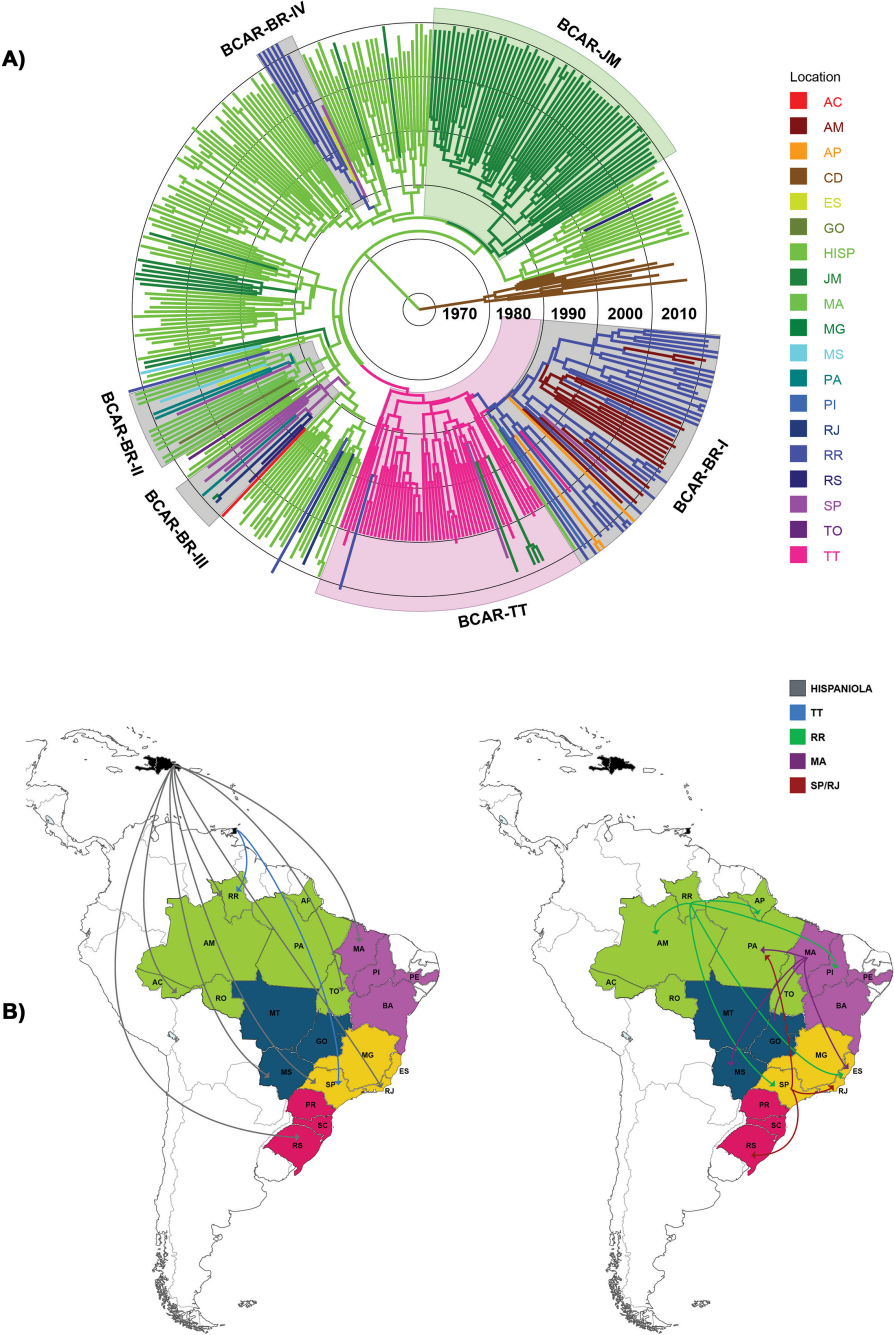
Spatiotemporal dynamics of dissemination of HIV-1 B_CAR_ clades circulating in the Caribbean and Brazil. A) Time-scaled Bayesian MCMC tree of *pol* PR/RT sequences of HIV-1 B_CAR_ lineages from Brazil (n = 97) and the Caribbean (*n* = 258), and subtype D reference sequences (*n* = 10) from the Democratic Republic of Congo. Branches are colored according to the most probable location state of their descendent nodes as indicated in the legend at right. Colored boxes indicate the positions of major B_CAR_ clades detected in Brazil, Jamaica and Trinidad and Tobago. Branch lengths are depicted in units of time (years). The tree was automatically rooted under the assumption of a relaxed molecular clock. B) Lines between locations represent branches in the Bayesian MCC tree along which location transitions occurred and were colored according to the location of origin (see the legend at left). Maps were created from templates obtained from d-maps.com (Caribbean: http://d-maps.com/carte.php?numcar=1389&lang=en; South America: http://d-maps.com/carte.php?numcar=2313&lang=en; and Brazil: http://d-maps.com/carte.php?numcar=4843&lang=en). AC: Acre; AM: Amazonas; AP: Amapá; CD: Democratic Republic of Congo; ES: Espírito Santo; GO: Goiás; HISP: Hispaniola; JM: Jamaica; MA: Maranhão; MG: Minas Gerais; MS: Mato Grosso do Sul; PA: Pará; PI: Piauí; RJ: Rio de Janeiro; RR: Roraima; RS: Rio Grande do Sul; SP: Sao Paulo; TO: Tocantins; TT: Trinidad and Tobago.

The HIV-1 B_CAR_ *pol* sequences with known sampling date from Brazil here identified (*n* = 97) were classified into 15 discrete geographic locations according to the sampling state (S2 Table). Brazilian B_CAR_ sequences were combined with B_CAR_ *pol* sequences from the most widely sampled Caribbean islands (Hispaniola, Jamaica and Trinidad and Tobago) previously identified [9,12], and with subtype D *pol* sequences from the Democratic Republic of Congo (DRC) (*n* = 10) that was pointed as the most probable source location of subtype B strain introduced in the Americas [8] and subsequently subjected to Bayesian phylogeographic reconstructions. The root location of the HIV-1 subtype B ancestor was most probably placed in the island of Hispaniola (Dominican Republic/Haiti) (posterior state probability [*PSP*] = 0.92) during the 1960s (Fig 3A and Table 2), consistent with previous findings [8,9]. The subtype B was then independently disseminated from Hispaniola to Trinidad and Tobago and Jamaica around the early 1970s, where seeded secondary outbreaks that resulted in the origin of the non-pandemic subclades B_CAR-TT_ and B_CAR-JM_ previously described [8,9]. This Bayesian analysis also indicates that B_CAR_ strains were disseminated at multiple times from Hispaniola (*n* = 11) and Trinidad and Tobago (*n* = 3) to Brazil (Fig 3). Direct disseminations of B_CAR_ strains from Hispaniola to Brazilian states of the Southern (Rio Grande do Sul), Southeastern (Rio de Janeiro and Sao Paulo), Central-Western (Mato Grosso do Sul), Northeastern (Maranhão) and Northern (Acre, Roraima and Tocantins) regions were detected, as well as dissemination of the B_CAR-TT_ clade from Trinidad and Tobago to Roraima and Sao Paulo states. The Bayes factor tests for significant nonzero rates, however, support epidemiological linkage between Hispaniola and only a few Brazilian states (Acre, Tocantins and Sao Paulo) as well as between Trinidad and Tobago and Roraima (S3 Table).

**Table 2:** Bayesian T_MRCA_ estimates for major B_CAR_ clades from Brazil and the Caribbean.

Clade	T_MRCA_ Current study	T_MRCA_ Cabello *et al* (2014)	T_MRCA_ Gilbert *et al* (2007)
Subtype B	1969 (1964–1974)	1964 (1959–1969)	1966 (1962–1970)
B_CAR-TT_	1973 (1970–1976)	1969 (1966–1973)	1973 (1970–1976)
B_CAR-JM_	1974 (1970–1979)	1971 (1967–1975)	-
B_CAR-BR-I_	1978 (1975–1981)	-	-
B_CAR-BR-II_	1978 (1974–1982)	-	-
B_CAR-BR-III_	1979 (1974–1983)	-	-
B_CAR-BR-IV_	1982 (1977–1986)	-	-

The mean estimated evolutionary rate of the HIV-1 B_CAR_/D *pol* dataset was 2.1 x 10^−3^ substitutions/site per year (95% HPD 2.0 x 10^−3^–2.2 x 10^−3^ substitutions/site per year), whereas the corresponding median coefficient of rate variation was 0.31 (95% HPD: 0.27–0.35), supporting the selection of a relaxed molecular clock model.

### Dispersal pattern of the Brazilian HIV-1 B_CAR_ clades

Among the 14 B_CAR_ strains introduced into Brazil, four established onward transmission and originated the Brazilian clades here denominated B_CAR-BR-I_, B_CAR-BR-II_, B_CAR-BR-III_ and B_CAR-BR-IV_ (Fig 3A)_,_ that comprise 51%, 16%, 10% and 8% of the Brazilian B_CAR_ sequences used in this analysis, respectively. All Brazilian non-pandemic subtype B clades displayed a high support (*PP* > 0.80) with exception of B_CAR-BR-III_ (*PP* = 0.35). The clade B_CAR-BR-I_ comprises most B_CAR_ sequences detected in Roraima (79%) and all sequences detected in Amazonas. This clade seems to have arisen by the introduction of a B_CAR-TT_ strain into Roraima at around 1978 (Table 2), with later dissemination from Roraima to: Amazonas, Amapá, Piauí and Sao Paulo (Fig 3). The clade B_CAR-BR-II_ comprises all B_CAR_ sequences detected in the state of Maranhão. This clade seems to have arisen by the introduction of a B_CAR_ strain from the Hispaniola into Maranhão at around 1978 (Table 2) and the subsequent dissemination from Maranhão to: Pará, Goiás, Mato Grosso do Sul, Sao Paulo and Espírito Santo (Fig 3). The clade B_CAR-BR-III_ probably arose by the introduction of a B_CAR_ strain from Hispaniola into the state of Sao Paulo at around 1979 (Table 2) and from Sao Paulo it was disseminated to: Rio de Janeiro, Minas Gerais, Rio Grande do Sul and Pará (Fig 3). The clade B_CAR-BR-IV_ probably arose by the introduction of a B_CAR_ strain from Hispaniola into the state of Roraima at around 1982 (Table 2) and from Roraima it was disseminated to Sao Paulo and Espírito Santo (Fig 3). The Bayes factor tests for significant nonzero rates supports epidemiological linkage between most Brazilian locations pairs previously described (S3 Table). Of note, among the 10 Brazilian homosexual/bisexual men infected by B_CAR_ strains here identified, five branched within the clade B_CAR-BR-I_, three within the clade B_CAR-BR-II_, one within the clade B_CAR-BR-IV_, and the remaining one branched outside the major Brazilian clades.

## Discussion

This study demonstrates that B_CAR_ strains have been introduced at multiples times into Brazil and circulate in at least 16 out 21 Brazilian states here analyzed. Although subtype B epidemic in most Brazilian states is clearly dominated by the B_PANDEMIC_ clade, the non-pandemic B_CAR_ strains reach a significant prevalence in a few states from the Northern (Roraima = 41% and Amazonas = 14%) and Northeastern (Maranhão = 14%) regions. The prevalence of B_CAR_ strains detected in Roraima is comparable to that described in some northern South American countries (Suriname and French Guyana), and much higher than that estimated for other continental countries of the Americas [10].

Our phylogeographic analysis indicates that the islands of Hispaniola and Trinidad and Tobago were probably the major sources of B_CAR_ lineages introduced into Brazil, although direct epidemiological linkages between the Caribbean islands and several Brazilian states were not significantly supported. It is highly probable that Suriname, French Guyana and Guyana may have also played a crucial role in such dissemination process, acting as a staging post between the Caribbean islands and Brazil. Those South American countries displayed a high prevalence of B_CAR_ strains [10] and have maintained a high human flux with both Caribbean islands and some Northern and Northeastern Brazilian states, facilitated not only by the geographical proximity, but also by economical factors [33–40]. Of note, one of the B_CAR_ strains detected in Roraima was isolated from an individual from the Guianese city of Lethem, located at border with Roraima. Unfortunately, the number of subtype B *pol* (PR/RT) sequences from those South American countries currently available in public database is too small to obtain robust phylogeographic reconstructions of the viral migrations pathways in the northernmost South American region.

Irrespective of the precise location of the source, our phylogeographic analysis clearly showed that several Brazilian states from the Northern (Roraima, Acre and Tocantins), Northeastern (Maranhão), Southeastern (Rio de Janeiro and Sao Paulo), Central-Western (Mato Grosso do Sul) and Southern (Rio Grande do Sul) regions acted as an entry point of B_CAR_ strains. Most Brazilian individuals infected with B_CAR_ strains were heterosexual (65%), although the proportion of individuals infected by heterosexual (44%) and homosexual/bisexual (40%) contacts was roughly similar among men caring B_CAR_ strains. These results revealed that the B_CAR_ strains are being introduced into both heterosexual and MSM networks from different Brazilian states. Most introductions seem to have resulted in dead-end infections that were not further disseminated in the Brazilian population. Four B_CAR_ strains, however, established onward transmission in the Brazilian population and originated local non-pandemic subtype B clades here designated from B_CAR-BR-I_ to B_CAR-BR-IV,_ according to their relative prevalence.

Roraima not only display the highest prevalence of B_CAR_ strains among all Brazilian states, but was also pointed as the most probable source location of B_CAR-BR-I_ and B_CAR-BR-IV_ clades. The clade B_CAR-BR-I_ probably evolved from the clade B_CAR-TT_ circulating in Trinidad and Tobago, while the clade B_CAR-BR-IV_ was more closely related to B_CAR_ strains from the Hispaniola. The clade B_CAR-BR-I_ was successfully spread within Roraima and disseminated to Amazonas at multiple times, and also to Amapá, Piauí and Sao Paulo. The pervasive dissemination of the clade B_CAR-BR-I_ from Roraima into Amazonas is expected considering that these two neighboring states maintain a very intense population flux through the BR-174 highway that connects both states and is the only accession route by land to Roraima from Brazil. The clade B_CAR-BR-IV_ displayed a more restricted spread in Roraima, but was also disseminated over long distances reaching Sao Paulo and Espírito Santo.

The estimated median T_MRCA_ of clades B_CAR-BR-I_ (1978) B_CAR-BR-IV_ (1982) coincides with a period of fast population growth and increasing geographical accessibility in Roraima. The population in Roraima increased from 41.000 to nearly 220.000 inhabitants between 1970 and 1990 [41,42]. This population growth was fueled by the creation of incentives to immigration and the inauguration of important highways that gave access to large areas of the state, including some at the border with Guyana [41,42]. Many Brazilian migrants initially attracted by the rise of legal/illegal mining activities in Roraima later migrated to Guyana, and Brazil is (together with Suriname and Venezuela) one of the major migrants exporting countries to Guyana [33,37,38]. The economic crisis in Guyana also produced an increasing migration flux of Guyanese people to Roraima since the 1960s onwards, particularly to the neighboring district of Bonfim and the state capital Boa Vista [33,37,38]. These drastic changes in the demographic structure and population mobility may have fueled the introduction and dissemination of Guyanese B_CAR_ strains into Roraima.

Maranhão display the highest prevalence of B_CAR_ strains outside the Northern Brazilian region and was pointed as the most probable source location of clade B_CAR-BR-II_ at around 1978. This clade was disseminated within Maranhão and from this state to Pará, Goiás, Mato Grosso do Sul, Sao Paulo and Espírito Santo. Most people that migrate to Roraima during the 1970s and 1980s were from Maranhão [33,41,42], which creates a potential link for the direct dissemination of B_CAR_ strains from Roraima to Maranhão. The clade B_CAR-BR-II_, however, is not closely related to the clade B_CAR-BR-I_, supporting an independent origin. We propose that the clade B_CAR-BR-II_ probably arose by the introduction of a B_CAR_ strain from Suriname or French Guiana that host about 15.000 and 20.000 Brazilian immigrants, particularly from Maranhão, Amapá and Pará states [33–36,39,40]. Many of those immigrants are female sex workers and gold-diggers (populations typically associated with a high risk of acquisition of HIV) that come back to Brazil from time to time and may thus introduce new B_CAR_ strains in the Northern and Northeastern regions.

The high prevalence of B_CAR_ strains detected in Roraima and Maranhão correlates with an intense migratory flux to Northern South American countries, but that association was not observed in other Brazilian states. Many individuals from the Northern Brazilian states of Amapá and Pará have migrated to the French Guyana since the middle 1960s and the social conditions in the border region between Amapá and French Guyana are certainly favorable for the spread of HIV [33–36]. Despite this, we detected a low proportion (3–4%) of B_CAR_ strains and no evidence of direct viral migrations from the Caribbean into Amapá or Pará. The B_CAR_ sequences detected in Amapá branched within the clade B_CAR-BR-I_, and the B_CAR_ sequences detected in Pará branched within clades B_CAR-BR-II_ and B_CAR-BR-III_. Thus, the B_CAR_ strains circulating in Amapá and Pará probably originated from other Brazilian states, rather than from neighboring Caribbean countries.

The clade B_CAR-BR-III_ was the only Brazilian non-pandemic subtype B lineage that originates outside the Northern/Northeastern region. This clade was most probably introduced from the Caribbean into the state of Sao Paulo at around 1979 and from there it was disseminated to Rio de Janeiro, Minas Gerais, Rio Grande do Sul and Pará. A previous study conducted by our group indicates that this clade (formerly named B_CAR-BR-I_) was also disseminated from Brazil to Argentina [10]. Sao Paulo is a potential hub for introduction and dissemination of new HIV-1 strains because it hosts the largest Brazilian international airport as well as a large number of international visitors and immigrants [43]. Although these results point to the existence of a B_CAR_ lineage mostly circulating in the southern area of South America, this should be interpreted with caution because the low branch support of the clade B_CAR-BR-III_.

In summary, this study demonstrates that non-pandemic HIV-1 B_CAR_ strains have been introduced at multiple times from the Caribbean into Brazil and reach a significant prevalence in some states from Northern (Roraima and Amazonas) and Northeastern (Maranhão) regions. Several Brazilian states from all country regions acted as an entry point of B_CAR_ strains, but only a few B_CAR_ strains, particularly those introduced into Roraima and Maranhão, established local outbreaks of relative large size. The molecular epidemiological surveillance of HIV-infected individuals from Guyana, French Guiana, and Suriname as well as of mobile populations migrating between Brazil and those neighboring countries will be of paramount importance to reconstruct the precise dissemination routes of B_CAR_ strains in the northernmost region of South America.

## Supporting Information

S1 Table HIV-1 subtype B *pol* (PR/RT and RT) sequences from Brazil, the Caribbean, US and France used for ML phylogenetic analyses. ^a^Antigua and Barbuda (n = 4), Bahamas (n = 5), Dominica (n = 1), Grenada (n = 2), Montserrat (n = 1), Saint Lucia (n = 4) and Saint Vincent and the Grenadines (n = 4).
(PDF)

S2 Table HIV-1 B_CAR_ *pol* (PR/RT) sequences from Brazil and the Caribbean used for Bayesian phylogeographic analysis. ^a^Identified in a previous study [9]. ^b^Subtype D sequences from the Democratic Republic of Congo (DRC).
(PDF)

S3 Table Bayes factor (BF) rates of epidemiological links between Caribbean and Brazilian locations for dispersal of non-pandemic BCAR lineages. BF > 100 indicates decisive support, 30 ≤ BF ≤ 100 indicates very strong support, 10 ≤ BF ≤ 30 indicates strong support, and 6 ≤ BF ≤ 10 indicates substantial support for migration between locations.
(PDF)

## References

[1] Brazilian (2015) Ministry of Health. AIDS Epidemiological Bulletin (in Portuguese) January-June 2015; Ano IV, n° 01 Available at: http://wwwaidsgovbr/sites/default/files/anexos/publicacao/2015/58534/boletim_aids_11_2015_web_pdf_19105pdf.

[2] Brindeiro RM, Diaz RS, Sabino EC, Morgado MG, Pires IL, Brigido L, et al (2003) Brazilian Network for HIV Drug Resistance Surveillance (HIV-BResNet): a survey of chronically infected individuals. Aids 17: 1063–1069. 12700457 10.1097/00002030-200305020-00016

[3] Stefani MM, Pereira GA, Lins JA, Alcantara KC, Silveira AA, Viegas AA, et al (2007) Molecular screening shows extensive HIV-1 genetic diversity in Central West Brazil. J Clin Virol 39: 205–209. 17537671 10.1016/j.jcv.2007.04.012

[4] de Moraes Soares CM, Vergara TR, Brites C, Brito JD, Grinberg G, Caseiro MM, et al (2014) Prevalence of transmitted HIV-1 antiretroviral resistance among patients initiating antiretroviral therapy in Brazil: a surveillance study using dried blood spots. J Int AIDS Soc 17: 19042 10.7448/IAS.17.1.19042 25249214 PMC4172689

[5] Guimaraes ML, Marques BC, Bertoni N, Teixeira SL, Morgado MG, Bastos FI, et al (2015) Assessing the HIV-1 Epidemic in Brazilian Drug Users: A Molecular Epidemiology Approach. PLoS One 10: e0141372 10.1371/journal.pone.0141372 26536040 PMC4633026

[6] Pessoa R, Loureiro P, Esther Lopes M, Carneiro-Proietti AB, Sabino EC, Busch MP, et al (2016) Ultra-Deep Sequencing of HIV-1 near Full-Length and Partial Proviral Genomes Reveals High Genetic Diversity among Brazilian Blood Donors. PLoS One 11: e0152499 10.1371/journal.pone.0152499 27031505 PMC4816342

[7] da Costa CM, Costa de Oliveira CM, Chehuan de Melo YF, Delatorre E, Bello G, Couto-Fernandez JC (2016) High HIV-1 Genetic Diversity in Patients from Northern Brazil. AIDS Res Hum Retroviruses. 10.1089/AID.2016.0044 27091699

[8] Gilbert MT, Rambaut A, Wlasiuk G, Spira TJ, Pitchenik AE, Worobey M (2007) The emergence of HIV/AIDS in the Americas and beyond. Proc Natl Acad Sci U S A 104: 18566–18570. 17978186 10.1073/pnas.0705329104 PMC2141817

[9] Cabello M, Mendoza Y, Bello G (2014) Spatiotemporal dynamics of dissemination of non-pandemic HIV-1 subtype B clades in the Caribbean region. PLoS One 9: e106045 10.1371/journal.pone.0106045 25148215 PMC4141835

[10] Cabello M, Junqueira DM, Bello G (2015) Dissemination of nonpandemic Caribbean HIV-1 subtype B clades in Latin America. AIDS 29: 483–492. 10.1097/QAD.0000000000000552 25630042

[11] Junqueira DM, de Medeiros RM, Matte MC, Araujo LA, Chies JA, Ashton-Prolla P, et al (2011) Reviewing the history of HIV-1: spread of subtype B in the Americas. PLoS ONE 6: e27489 10.1371/journal.pone.0027489 22132104 PMC3223166

[12] Mendoza Y, Martinez AA, Castillo Mewa J, Gonzalez C, Garcia-Morales C, Avila-Rios S, et al (2014) Human Immunodeficiency Virus Type 1 (HIV-1) Subtype B Epidemic in Panama Is Mainly Driven by Dissemination of Country-Specific Clades. PLoS ONE 9(4):: e95360 10.1371/journal.pone.0095360 24748274 PMC3991702

[13] Dos Anjos Silva L, Divino F, da Silva Rego MO, Lima Lopes IG, Nobrega Costa CM, da Silva Pereira FC, et al (2016) HIV-1 Genetic Diversity and Transmitted Drug Resistance in Antiretroviral Treatment-Naive Individuals from Amapa State, Northern Brazil. AIDS Res Hum Retroviruses 32: 373–376. 10.1089/AID.2015.0280 26529282

[14] de Oliveira T, Deforche K, Cassol S, Salminen M, Paraskevis D, Seebregts C, et al (2005) An automated genotyping system for analysis of HIV-1 and other microbial sequences. Bioinformatics 21: 3797–3800. 16076886 10.1093/bioinformatics/bti607

[15] Thompson JD, Gibson TJ, Plewniak F, Jeanmougin F, Higgins DG (1997) The CLUSTAL_X windows interface: flexible strategies for multiple sequence alignment aided by quality analysis tools. Nucleic Acids Res 25: 4876–4882. 9396791 10.1093/nar/25.24.4876 PMC147148

[16] Posada D (2008) jModelTest: phylogenetic model averaging. Mol Biol Evol 25: 1253–1256. 10.1093/molbev/msn083 18397919

[17] Guindon S, Dufayard JF, Lefort V, Anisimova M, Hordijk W, Gascuel O (2010) New algorithms and methods to estimate maximum-likelihood phylogenies: assessing the performance of PhyML 3.0. Syst Biol 59: 307–321. 10.1093/sysbio/syq010 20525638

[18] Guindon S, Lethiec F, Duroux P, Gascuel O (2005) PHYML Online—a web server for fast maximum likelihood-based phylogenetic inference. Nucleic Acids Res 33: W557–559. 15980534 10.1093/nar/gki352 PMC1160113

[19] Anisimova M, Gascuel O (2006) Approximate likelihood-ratio test for branches: A fast, accurate, and powerful alternative. Syst Biol 55: 539–552. 16785212 10.1080/10635150600755453

[20] Rambaut A (2009) FigTree v1.4: Tree Figure Drawing Tool. Available: http://treebioedacuk/software/figtree/.

[21] Drummond AJ, Nicholls GK, Rodrigo AG, Solomon W (2002) Estimating mutation parameters, population history and genealogy simultaneously from temporally spaced sequence data. Genetics 161: 1307–1320. 12136032 10.1093/genetics/161.3.1307 PMC1462188

[22] Drummond AJ, Rambaut A (2007) BEAST: Bayesian evolutionary analysis by sampling trees. BMC Evol Biol 7: 214 17996036 10.1186/1471-2148-7-214 PMC2247476

[23] Suchard MA, Rambaut A (2009) Many-core algorithms for statistical phylogenetics. Bioinformatics 25: 1370–1376. 10.1093/bioinformatics/btp244 19369496 PMC2682525

[24] Drummond AJ, Ho SY, Phillips MJ, Rambaut A (2006) Relaxed phylogenetics and dating with confidence. PLoS Biol 4: e88 16683862 10.1371/journal.pbio.0040088 PMC1395354

[25] Drummond AJ, Rambaut A, Shapiro B, Pybus OG (2005) Bayesian coalescent inference of past population dynamics from molecular sequences. Mol Biol Evol 22: 1185–1192. 15703244 10.1093/molbev/msi103

[26] Hue S, Pillay D, Clewley JP, Pybus OG (2005) Genetic analysis reveals the complex structure of HIV-1 transmission within defined risk groups. Proc Natl Acad Sci U S A 102: 4425–4429. 15767575 10.1073/pnas.0407534102 PMC555492

[27] Zehender G, Ebranati E, Lai A, Santoro MM, Alteri C, Giuliani M, et al (2010) Population dynamics of HIV-1 subtype B in a cohort of men-having-sex-with-men in Rome, Italy. J Acquir Immune Defic Syndr 55: 156–160. 10.1097/QAI.0b013e3181eb3002 20703157

[28] Chen JH, Wong KH, Chan KC, To SW, Chen Z, Yam WC (2011) Phylodynamics of HIV-1 subtype B among the men-having-sex-with-men (MSM) population in Hong Kong. PLoS ONE 6: e25286 10.1371/journal.pone.0025286 21966483 PMC3178636

[29] Lemey P, Rambaut A, Drummond AJ, Suchard MA (2009) Bayesian phylogeography finds its roots. PLoS Comput Biol 5: e1000520 10.1371/journal.pcbi.1000520 19779555 PMC2740835

[30] Ferreira MAR, M.A. S (2008) Bayesian analysis of elapsed times in continuous-time Markov chains. Canadian Journal of Statistics 26: 355–368.

[31] Rambaut A, Drummond A (2007) Tracer v1.6. Available: http://treebioedacuk/software/tracer/.

[32] Bielejec F, Rambaut A, Suchard MA, Lemey P (2011) SPREAD: spatial phylogenetic reconstruction of evolutionary dynamics. Bioinformatics 27: 2910–2912. 10.1093/bioinformatics/btr481 21911333 PMC3187652

[33] Leonardi V (2000) Fronteiras Amazonicas do Brasil: saúde e história social (in Portuguese). Brasilia: Paralelo 15; Sao Paulo: Marco Zero.

[34] Arouck R (2000) Brasileiros na Guiana francesa. Novas migrações internacionais ou exportação de tensões sociais na Amazônia? [in Portuguese]. Lusotopie: 67–78. Available: http://wwwlusotopiesciencespobordeauxfr/arouckpdf.

[35] Soares CL, de Souza Oliveira B, de Souza Pinto MJ (2011) Trabalhadores brasileiros na Guiana Francesa: entre a invisibilidade e o desemprego (in Portuguese). PRACS: Revista de Humanidades do Curso de Ciências Sociais da UNIFAP 4: 129–142. Available: https://periodicosunifapbr/indexphp/pracs/article/view/407/n4Christiannypdf.

[36] Martins CC (2011) Migração transfronteriça na Amazônia: Brasileiros na Guiana Francesa (in Portuguese). Anais do III Simpósio de Pós-Graduação em Relações Internacionais do Programa “San Tiago Dantas” (UNESP, UNICAMP e PUC/SP) Available: http://wwwunespbr/santiagodantassp.

[37] Pereira MC (2006) Processos migratórios na fronteira Brasil-Guiana (in Portuguese). Estudos Avançados 20: 209–219. Available: http://wwwscielobr/pdf/ea/v20n57/a16v2057pdf.

[38] Corbin HP (2007) Brazilian migration to Guyana as a livelihood strategy: a case study approach. Available: http://wwwrepositorioufpabr/jspui/bitstream/2011/1966/1/Dissertacao_BrazilianMigrationGuyanapdf.

[39] de Theije M, Heemskerk M (2009) Moving Frontiers in the Amazon: Brazilian Small-Scale Gold Miners in Suriname. European Review of Latin American and Caribbean Studies: 5–25. Available: http://www.cedla.uva.nl/50_publications/pdf/revista/87RevistaEuropea/87-DETHEIJE&HEEMSKERK-ISSN-0924-0608.pdf.

[40] Heemskerk M, Duijves C (2014) Suriname Migration Profile: a study on emigration from, and immigration into Suriname International Organization for Migration (IOM) Accessed http://wwwmigration-eu-laceu/documents/keydocs/MP_Surinam/MP_Surinamepdf.

[41] Diniz AMA, dos Santos RO (2005) O vertiginoso crescimento populacional de Roraima e seus impactos socioambientais (in Portuguese). Caderno de Geografia 15: 23–44.

[42] Vale ALF (2006) Imigração de nordestinos para Roraima (in Portuguese). Estudos Avançados 20: 255–261. Available: http://wwwscielobr/pdf/ea/v20n57/a19v2057pdf.

[43] Amaral EF, Fusco W (2005) Shaping Brazil: The Role of International Migration. The Online Journal of the Migration Policy Institute. Available: http://www.migrationpolicy.org/article/shaping-brazil-role-international-migration.

