# Supplementary material for: High Prevalence and Onward Transmission of Non-Pandemic HIV-1 Subtype B Clades in Northern and Northeastern Brazilian Regions
Source: PLoS One. 2016 Sep 7;11(9):e0162112. doi: 10.1371/journal.pone.0162112 (PMC5014447; doi:10.1371/journal.pone.0162112)
Supplement: S1 Table — aAntigua and Barbuda (n = 4), Bahamas (n = 5), Dominica (n = 1), Grenada (n = 2), Montserrat (n = 1), Saint Lucia (n = 4) and Saint Vincent and the Grenadines (n = 4). (PDF) [file pone.0162112.s001.pdf]

S1 Table. HIV-1 subtype B *pol* (PR/RT) sequences from Brazil, the Caribbean, US and France used for ML phylogenetic analyses.

**Subset**	**Country**	**State**	***N***	**Sampling time**
A	Brazil	SP	1,205	1998-2010
B		ES/GO/MG/MS/MT/ PR/RJ/RS/SC	767	1997-2010
C		AC/AM/AP/BA/MA/ PA/PE/PI/RO/RR/TO	710	2008-2013
A/B/C	Dominican Republic		61	2005-2010
	Haiti		8	2004-2005
	Jamaica		62	2005-2010
	Trinidad and Tobago		48	2000-2003
	Other Caribbean countries[a]		21	2000-2004
	US		165	1997-2009
	France		135	1985-2008

[a] Antigua and Barbuda (*n* = 4), Bahamas (*n* = 5), Dominica (*n* = 1), Grenada (*n* = 2), Montserrat (*n* = 1), Saint Lucia (*n* = 4) and Saint Vincent and the Grenadines (*n* = 4).
