# Supplementary material for: High Prevalence and Onward Transmission of Non-Pandemic HIV-1 Subtype B Clades in Northern and Northeastern Brazilian Regions
Source: PLoS One. 2016 Sep 7;11(9):e0162112. doi: 10.1371/journal.pone.0162112 (PMC5014447; doi:10.1371/journal.pone.0162112)
Supplement: S2 Table — aIdentified in a previous study [9]. bSubtype D sequences from the Democratic Republic of Congo (DRC). (PDF) [file pone.0162112.s002.pdf]

**S2 Table.** HIV-1 $B_{CAR}$ *pol* (PR/RT) sequences from Brazil and the Caribbean used for Bayesian phylogeographic analysis.

| Country | Region | State | Location | *N* | Sampling date |
|---|---|---|---|---|---|
| Brazil | Northern | AC | AC | 1 | 2010 |
| | | AM | AM | 15 | 2009-2011 |
| | | AP | AP | 3 | 2013 |
| | | PA | PA | 3 | 2010 |
| | | RR | RR | 42 | 2010-2013 |
| | | TO | TO | 1 | 2008 |
| | Northeastern | MA | MA | 10 | 2012 |
| | | PI | PI | 1 | 2011 |
| | Central-Western | GO | GO | 1 | 2008 |
| | | MS | MS | 2 | 2008-2010 |
| | Southeastern | ES | ES | 2 | 1997 |
| | | MG | MG | 1 | 2009 |
| | | RJ | RJ | 3 | 2004-2009 |
| | | SP | SP | 9 | 1999-2008 |
| | Southern | RS | RS | 3 | 1998-2005 |
| Dominican Republic[a] | | - | HISP | 123 | 2003-2011 |
| Haiti[a] | | - | HISP | 12 | 2004-2005 |
| Jamaica[a] | | - | JM | 73 | 2005-2010 |
| Trinidad and Tobago[a] | | - | TT | 50 | 2000-2003 |
| DRC[b] | | - | CD | 10 | 1983-2007 |

[a] Identified in a previous study [9]. [b] Subtype D sequences from the Democratic Republic of Congo (DRC).
