# Supplementary material for: High Prevalence and Onward Transmission of Non-Pandemic HIV-1 Subtype B Clades in Northern and Northeastern Brazilian Regions
Source: PLoS One. 2016 Sep 7;11(9):e0162112. doi: 10.1371/journal.pone.0162112 (PMC5014447; doi:10.1371/journal.pone.0162112)
Supplement: S3 Table — BF > 100 indicates decisive support, 30 ≤ BF ≤ 100 indicates very strong support, 10 ≤ BF ≤ 30 indicates strong support, and 6 ≤ BF ≤ 10 indicates substantial support for migration between locations. (PDF) [file pone.0162112.s003.pdf]

S3 Table. Bayes factor (BF) rates of epidemiological links between Caribbean and Brazilian locations for dispersal of non-pandemic B_{CAR} lineages.

**Regions**	**Locations**	**BF**[a]
Caribbean-Caribbean	HISP-TT	36
	HISP-JM	73,321
	TT-JM	16
Caribbean-Brazil	HISP-SP	149
	HISP-AC	10
	HISP-TO	23
	TT-RR	73,321
	Others	<3
Brazil-Brazil	RR-AM	73,321
	RR-AP	287
	RR-SP	188
	RR-PI	18
	MA-GO	8
	MA-PA	3
	MA-SP	57
	SP-ES	28
	SP-GO	4
	SP-MS	11
	SP-PA	28
	SP-RJ	38
	SP-RS	145
	RJ-MG	334
	Others	<3

[a] BF > 100 indicates decisive support, 30 ≤ BF ≤ 100 indicates very strong support, 10 ≤ BF ≤ 30 indicates strong support, and 6 ≤ BF ≤ 10 indicates substantial support for migration between locations.
